# Young Women

**Published:** 1875-06

**Authors:** 


Young women should be taught how to work, and be brought up with a willingness to work, whenever or wherever circumstances demand it. They should be made to regard it as a solemn duty to assist their husbands in the battle of life; and to think it no hardship to accept of "love in a cottage," where love and work must go hand in hand.

---

It begins to be seen that the poor are only they who feel poor, and poverty consists in feeling poor. The rich, as we reckon them—and among them the very rich—in a true scale, would be found very indigent and ragged. The really great make us feel, first of all, the indifference of circumstances. They call into activity the higher perceptions, and subdue the low habits of comfort and luxury; but the higher perceptions find their objects everywhere; only the low habits need palaces and banquets.

---

Young married life should begin to-day where it did fifty years ago, in a home of one room, if need be, and by mutual labor and economy, grow up into more ample accommodations.

